# Identification and validation of the phosphorylation sites on Aristaless-related homeobox protein

**DOI:** 10.1042/BSR20194513

**Published:** 2020-07-15

**Authors:** Xiuyu Shi, Wenbo Lin, Xiang Gao, Wen Xie, Jeffrey A. Golden, Tao Tao

**Affiliations:** 1 State Key Laboratory of Stress Cell Biology, School of Life Sciences, Xiamen University, Xiamen, Fujian 361005, China; 2 Department of Pathology, Brigham and Women’s Hospital, Harvard Medical School, Boston, MA 02115, U.S.A.; 3 School of Pharmaceutical Sciences, Xiamen University, Xiamen, Fujian 361005, China

**Keywords:** Aristaless-related homeobox, Cortex development, Mass Spectrometry, phosphorylation

## Abstract

The Aristaless-related homeobox protein (ARX) is a transcription factor expressed in the developing forebrain, skeletal muscle, pancreas, testis, and a variety of other tissues. It is known to have context-dependent transcriptional activator and repressor activity, although how it can achieve these opposing functions remains poorly understood. We hypothesized phosphorylation status might play a role in pivoting ARX between functioning as an activator or repressor. To gain further mechanistic insight as to how ARX functions, we identified multiple phosphorylation sites on ARX. We further established PKA as the kinase that phosphorylates ARX at least at Ser^266^ in mice. Two other kinases, CK2α and CDK4/cyclin D1, were also identified as kinases that phosphorylate ARX *in vitro*. Unexpectedly, phosphorylation status did not change either the nuclear localization or transcriptional function of ARX.

## Introduction

Post-translational modification of proteins is an essential cellular process that influences the protein localization, function, and degradation. Phosphorylation is one of the most common modifications that can take place at one or multiple sites, and at different rates along the protein to modulate their functions [1–3]. For example, the activity of transcription factors could be regulated by phosphorylation and many transcriptional factors have been shown to be phosphorylated at multiple sites by one or multiple kinases [4,5]. Sp1, a well-known bi-functional (activator and inhibitor) transcription factor, can be phosphorylated by ERK [6], PKC [7], CK2 [8], CDK1 [9], as well as many other kinases. Among these, phosphorylation of Sp1 by ERK, PKC, and CK2 increased its DNA binding activity, whereas phosphorylation by CDK1 decreased the activity [10]. Furthermore, in smooth muscle cells, Sp1 functions as a transcriptional activator after phosphorylation by ERK, but is switched to a repressor of platelet-derived growth factor receptor α (PDGFR-α) transcription after phosphorylation triggered by fibroblast growth factor 2 (FGF-2) [11].

Similar to Sp1, the gene product of *Aristaless-related homeobox* protein (*ARX/Arx*) is also a bi-functional transcription factor [12,13]. ARX is a paired-like transcription factor plays essential roles in brain, pancreas, skeletal muscle, and gonad development in both human and mice [14–18] The structure of ARX is highly conserved across species, including the DNA binding paired-like homeobox domain, three nuclear localization sequences (NLSs), four polyalanine tracts of variable length, an N-terminal octapeptide domain (OD) and a C-terminal Aristaless domain (AD) [19,20]. Mutations associated with these domains often interfere with the function of ARX and have been implicated in a broad spectrum of neurodevelopmental disorders [21]. Additionally, disruption to the location of ARX may alter its function in a cell. Previous work from our lab implicated an essential role for importins in the cellular localization of ARX [20]. Knowing the shuttling of proteins by importins is often phosphorylation dependent [22], we postulated that the phosphorylation of ARX would be necessary for its cellular localization and potentially function(s).

A previous study revealed three phosphorylation sites (S37, S67, and S174) on ARX (human) and confirmed S174 as the major protein kinase C α (PKCα) phosphorylation target [23]. Additionally, overexpression of ARX with the phospho-null mutation of these sites changes the transcriptomes of alpha-TC cells ( a pancreatic cell line) [23]. However, many aspects regarding ARX phosphorylation remain unknown, especially whether phosphorylation effects the function of ARX *in vivo*.

In the current study, we report the identification of multiple ARX phosphorylation sites and sought to determine their functional significance. We also suggest that the phosphorylation of ARX could be achieved through several different kinases. But unexpectedly, the ARX localization and functions we assayed, including radial migration of the progenitor cells to the cortex, were not impacted by the phosphorylation at the identified sites. Nonetheless, the phosphorylation of ARX could still play roles via other sites not identified in the present study, at specific time points, in the different tissues, or even in a stress response not tested in our studies.

## Materials and methods

All studies were performed using the mouse *Arx* sequence. While highly homologous to the human sequence, minor differences do exist, including at least one identified phosphorylation site.

### Mice

All mouse studies were performed in accordance with the guidelines in the Guide for the Care and Use of Laboratory Animals of the National Institutes of Health. The experiments were approved by and performed in Brigham and Women’s Hospital/Harvard Medical School, the Center for Comparative Medicine (protocol number: 2016N000244). Floxed *Arx* mice (*Arx^flox/flox^*) [24] were mated with *Ai14*^*hom*^ mice (a Cre drove TdTomato reporter mice, from The Jackson Laboratory, stock #: 007914) to generate the *Arx^flox/flox^ Ai14*^*hom*^ and *Arx^flox/Y^ Ai14*^*hom*^ mice. Timed-pregnant dams were anesthetized during the surgeries using isoflurane followed the protocol above, sacrificed by cervical dislocation, and the embryos were immediately harvested for histological analyses.

### Gel shift assay

Gel shift assay was performed as previously described with minor modifications [25]. HEK 293T cells were grown in Dulbecco’s Modified Eagle’s Medium (Invitrogen, Life Technologies, Grand Island, NY) containing 10% fetal bovine serum in 3.5 cm plates and transfected with *Arx* expression vectors using polyethyleneimine (PEI) (Polysciences). After a brief sonication in 150 μl of phosphatase buffer (50 mM Tris [pH 8.0], 1 mM MgCl_2_, 0.1 mM ZnCl_2_, 0.1% sodium dodecyl sulfate [SDS], 10% glycerol, 1 mM dithiothreitol [DTT], 0.1 mM phenylmethylsulfonyl fluoride [PMSF]), the cells were centrifuged at 12000 rpm for 10 min. Supernatants (50 μl) were incubated with 10 U of calf intestinal alkaline phosphatase (CIAP) (Takara) for 30 min at 30°C. As a control, the same reaction was performed in the presence of 60 mM Na_2_HPO_4_, which competed for the phosphatase reaction. The total protein concentration of the supernatants was determined by the BCA Protein Assay Kit (Pierce); equal amounts of protein were used for the gel shift assay. ARX protein expression was detected by the relevant tag antibody. The gel shift assay for the treatment with Calyculin A (CA, Cell Signaling Technology) was similar as the CIAP treatment experiment: after 24 h of the transfection, cells were treated with 100 nM CA for 30 min before lysis, a DMSO (Sigma–Aldrich) treated group was used as the control.

### LC-MS/MS and analysis of spectra

HEK 293T cells transfected with *pcDNA3.1-FLAG-Arx* were treated with or without 100 nM CA for 30 min before lysis in lysis buffer (20 mM Tris/HCl [pH 7.5], 150 mM NaCl, 1 mM EDTA, 1% Triton X-100) and precleared with Protein A/G Agarose (Pierce) for 1 h before incubation with anti-FLAG antibody (Sigma) overnight at 4°C, followed by adding Protein A/G Agarose beads for another 3 h before washing five times with washing buffer (same as lysis buffer except salt concentration was increased to 300 mM). Immunoprecipitates were boiled in SDS loading buffer and ran on an SDS/PAGE gel, followed by Coomassie Blue staining. Bands corresponding to ARX were in-gel digested with trypsin. LC-MS/MS was next performed with Q Exactive™, and the data were analyzed with Proteome Discoverer version 2.1 by Xiang Gao Lab from the School of Pharmaceutical Sciences, Xiamen University.

### *In vitro* kinase assay

The Sf9 cells were cultured in Sf-900 II SFM (Thermo Fisher Scientific) and infected with a recombinant baculovirus expressing ARX^FLAG^ for 48 h at a multiplicity of infection of 10. The cells (2 × 10^6^ cells/ml, 100 ml) were harvested, resuspended in 15 ml of buffer A (20 mM HEPES [pH 7.4], 150 mM NaCl, 5% glycerol, 1% Triton X-100, 0.5% Deoxycholic acid, 0.1% SDS, 0.5 mM EDTA, 1.5 mM MgCl_2_, and protease inhibitors), and lysed by sonication (Branson Digital Sonifier 250, 10 on/off cycles (2 s/2 s) of sonication at 15% output). The lysate was centrifuged at 13000 ***g*** for 15 min at 4°C, and the supernatant was incubated with ANTI-FLAG® M2 Affinity Gel for 3 h at 4°C. The beads were sequentially washed with buffer A containing 0.3, 0.6, 1.0, 0.6, 0.3, 0.15M NaCl. Finally, the beads were washed in buffer B (25 mM Tris-Cl, pH 7.4, 150 mM NaCl, 0.1% Triton X-100), and ARX^FLAG^ was eluted in buffer B containing FLAG peptide (100 μg/ml). Eluted ARX^FLAG^ was subjected to SDS/PAGE, and the protein concentration was measured by comparing intensities of Coomassie-stained bands with those of BSA control. *In vitro* kinase assays with purified ARX protein were performed using the HotSpot platform from Reaction Biology Co. (RBC).

### Heterokaryon assay

HeLa cells growing in Dulbecco’s Modified Eagle’s Medium (Invitrogen, Life Technologies, Grand Island, NY) containing 10% fetal bovine serum in six-well plates were transfected with *pCIG-Arx^myc^-IRES-eGFP*, expressing wildtype (WT) or the ARX gain/loss of function mutant (mut) constructs. After 24 h, HeLa cells (4 × 10^4^ cells/well) were resuspended and co-cultured with the same number (4 × 10^4^/well/cell line) of NIH3T3 cells in eight-well chamber slide (BD Falcon™) for 18 h. Cycloheximide (CHX) was added (100 μg/ml) for 3 h before inducing heterokaryons. Fifty percent (w/v) PEG 4000 solution was added for 2 min at room temperature to induce heterokaryon formation. Subsequently, the cells were extensively washed, and further incubated at 37°C in the presence of CHX. The cells were fixed after 4 h with 4% paraformaldehyde (PFA) for subsequent immunostaining.

### Immunofluorescence, fluorescence microscopy, and image analysis

Fixed cells were permeabilized with 0.1% Triton X-100 in phosphate buffer saline for 5 min. Cells expressing myc-tagged ARX were also incubated with an anti-Myc antibody (1:3000, CST) for 1 h at room temperature and subsequently labeled with an Alexa 594-conjugated (1:200, Invitrogen) secondary antibody for 1 h at room temperature. All cells or heterokaryons were incubated with DAPI (1:10000, Invitrogen) for 10 s and subsequently imaged as below. Immunofluorescence for tissue was performed as previously reported [26]. Briefly, embryos were collected, fixed overnight in cold 4% PFA, washed at 4°C in PBS, cryoprotected overnight at 4°C in PBS containing 30% sucrose, and frozen in OCT (Tissue-Tek). Fifteen-micrometer-sections were cut for subsequent immunostaining. A minimum of three sections, from at least three embryos, were examined in the mouse studies for each construct. Slices were incubated with anti-RFP antibody (1:500, MBL International) overnight at 4°C and subsequently labeled with Alexa 594-conjugated (1:300, Invitrogen) secondary antibody for 1 h at room temperature.

All images were taken using a fluorescence microscope on a Zeiss Observer Z1 inverted microscope running Zeiss Zen Pro software driving a Hamamatsu ORCA-Flas 4.0 camera (Carl Zeiss, Oberkochen, Germany). Cell numbers were counted in Photoshop (Adobe) CC 2018.

### Electrophoretic mobility shift assays

Sequences corresponding to amino acids 220–392 of ARX^WT/mut^ were amplified from *pEGFP-C1-Arx*^*WT/mut*^ as described above and then cloned into the vector of pGEX-4T-2 (GE Healthcare Life Sciences). GST-ARX (220–392)^WT/mut^ protein was expressed in *Escherichia coli* BL21 competent cells (Invitrogen), and its expression was induced by the addition of 0.2 mM IPTG to late logarithmic cultures (OD = 0.6) for 4 h at 25°C. Cells were then harvested, resuspended in bacteria lysis buffer (50 mM Tris/HCl [pH 8.0], 200 mM NaCl, 200 mM EDTA, 10% Glycerol, 1 mM DTT, 1 mM PMSF, 1 mg/ml lysozyme) and disrupted by sonication. After centrifugation, the supernatants were applied to Glutathione Sepharose 4B beads (GE Healthcare Life Sciences), and the beads were washed with PBS. The GST fusion protein was finally eluted with 30 mM reduced glutathione in 50 mM Tris-Cl, pH 8.0.

Electrophoretic mobility shift assays (EMSAs) were performed as previously described with slight modifications [24]. The purified ARX protein truncation was applied to 6% polyacrylamide gel with double-stranded oligonucleotides, SIX1, F (5′-TCTTAACATTAAGGTAATTAAATATGAGCTCAC-3′)/SIX1, R (5′-GTGAGCTCATATTTAATTACCTTAATGTTAAGA-3′) labeled by biotin (Sangon Biotech). To detect the DNA/ARX complex, we utilized the LightShift® Chemiluminescent EMSA kit (Thermo Scientific).

### Antibodies

All tag antibodies are described above. The ARX Ser^266^ site-specific phosphorylation antibody (0.68 mg/ml, 1:1000 for immunoblot experiments) was generated by ABclonal Technology (antigen sequence: CLKEPRRCs^p^VATTGTV). The α-ARX (R&D Systems) antibody was used to detect WT ARX on Western blots.

### Luciferase assay

HEK293T cells were plated (4 × 10^5^ cells/well) in a 12-well culture plate the day before transfection. Cells were transfected with 200 ng luciferase reporter construct (4× TAATTA-TK-Luciferase, Lmo1-TK-Luciferase, or Ebf3-TK-Luciferase) or 400 ng TOP-flash luciferase reporter constructs, 50 ng pRL-TK *Renilla* luciferase as an internal control, and 400 or 100 ng (for TOP-flash assay) ARX expression construct or one of the ARX mutant constructs. The cells were split into a poly-d-lysine coated (Sigma) 96-well plate (4 × 10^4^ cells/well) 6 h post-transfection and incubated for an additional 16 h. For TOP-flash assays, the cells were treated with recombinant Wnt3a (R&D systems) at 100 ng/ml for 6 h. The luciferase assays were performed using the Dual-Glo Luciferase Assay System (Promega) according to the manufacturer’s protocol. The luciferase reporter activity is presented as relative light units (RLUs). Each experiment was performed in triplicate.

### *In utero* electroporation

*In utero* electroporation (IUEP) was performed as described previously on embryonic day 13.5 (E13.5) [12]. Specifically, a *Nestin:Arx^WT^*^ or^ *^8SA^-IRES-Cre* construct were injected into the ventricle of the *Arx^flox/flox^ Ai14*^*hom*^ or *Arx^flox/Y^ Ai14*^*hom*^ mouse embryos, followed by trans-uterine electroporation. The electroporated cells express TdTomato along with the abrogation of the endogenous ARX. Four days after IUEP, the dams were sacrificed, and the E17.5 embryonic brains were collected. The brains were fixed overnight in 4% PFA and prepared for cryosectioning (15 μm) as described [27]. Sections from the similar level of three brains for each genotype were used for quantification. All sections were imaged on the Zeiss Observer Z1 inverted microscope described above.

### Statistical analysis

All statistical analyses were done in GraphPad Prism 7 software using the two-tailed unpaired *t* test. All graphs are plotted as mean ± the standard error of the mean (SEM).

## Results

### ARX is phosphorylated in HEK 293T cells

**Figure 1:**
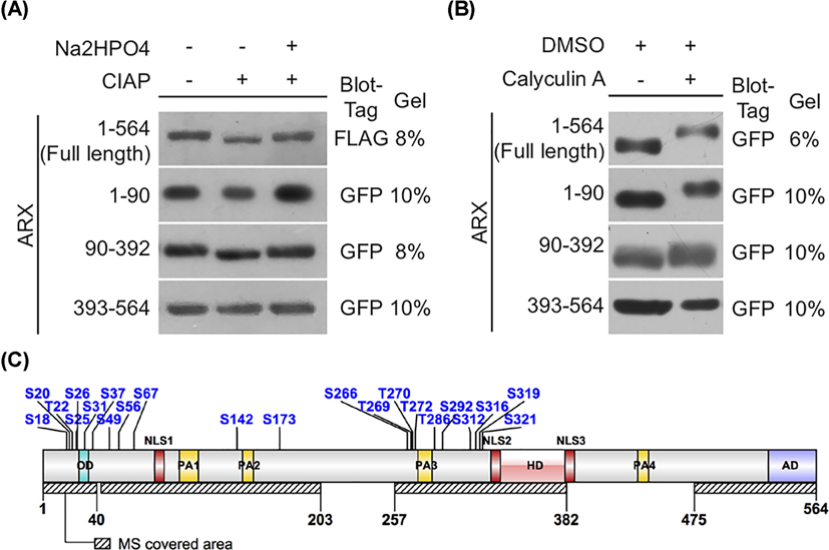
Identification of ARX phosphorylation status and sites Western blots of 293T cells transiently transfected with the expression vector encoding full-length *Arx* or various truncation mutations of *Arx* (as indicated), each fused to FLAG or eGFP tag as indicated. (**A**) Cell lysates were incubated with or without CIAP (as indicated). Faster migrating (lower) bands represent dephosphorylated ARX protein. Inhibition of the CIAP reaction with Na_2_HPO_4_ abolished the shift. (**B**) The ARX bands migrate significantly slower (higher bands) after treatment with 100 nM CA for 30 min. (**C**) A schematic of the phosphorylation sites identified by MS on ARX. Although variation exists for the area covered in each single experiment, over 60% coverage (dashed bars) was present across studies (Abbreviations: HD, homeodomain; MS, mass spectrometry; PA, poly-alanine).

Phosphorylation is known to regulate the localization and the function of many transcription factors [3,4]. Given the diverse functions of ARX [12], it is reasonable to consider phosphorylation as an essential regulator of its functions and possibly cellular localization. To determine if ARX is phosphorylated, cell lysates were assayed before and after the CIAP (dephosphorylation) treatment, as previously described [25]. ARX showed increased electrophoretic mobility after dephosphorylation (Figure 1A). Moreover, the band shift was entirely or partially inhibited by the addition of excess phosphate from Na_2_HPO_4_ (a competitive inhibitor) (Figure 1A).

Conversely, treatment with CA, a protein phosphatase1 (PP1) and PP2A inhibitor [28,29], resulted in an altered electrophoretic mobility shift of ARX reflecting the stabilized phosphorylation (Figure 1B). To refine the mapping of ARX phosphorylation, we engineered expression constructs consisting of amino acids 1–90, 90–392, and 393–564. Interestingly, both CIAP and CA treatments resulted in electrophoretic mobility shifts for the 1–90 and 90–392 fragments. However, no shift was observed with the 393–564 fragment (Figure 1A,B). These data suggest that ARX phosphorylation sites reside primarily in the N terminal two-thirds of ARX, or the phosphorylation of the C-terminal is highly sensitive to the 3-dimensional structure that is disrupted when truncated.

### Twenty-two phosphorylation sites of ARX were identified by mass spectrometry

To further define the phosphorylation sites in ARX, we purified FLAG-tagged ARX protein from CA-treated or control 293T cells by immunoprecipitation (IP) and evaluated the purified protein by Mass Spectrometry (MS). A total of 22 phosphorylated amino acid residues were detected in the control and CA groups (Figure 1C, Table 1). The Ser^173^ (S173), S266, S292, S312, S316, S319, and S321 sites were noted to be in close proximity to NLS 2 (NLS2) and thus potential candidate sites for participating in the localization of ARX to the nucleus (Figure 1C). Furthermore, S292, S316, and S319 have been identified in all three repeat MS experiments with high confidence and adjacent to the homeodomain, NLS2, and NLS3, making them strong candidates for regulating ARX localization or function. Somewhat surprisingly, most of these sites did not localize to any of the known functional domains. S49, S56, S173, S266, Thr^269^ (T269), T270, T272 were detected only in the CA-treated group, although the S49, S56, and S173 sites lacked coverage in the control groups, making the interpretation of these sites uncertain (Figure 1C, Supplementary Figure S1). In contrast, S18, S20, S22, S25, S26, S31, S142, and T286 were detected only in the control group. Ser^173^ was of particular interest as it was reported to be phosphorylated by PKC [30,23]. Curiously, no amino acids in the C-terminus, between aa 322 and 564, were found to be phosphorylated, consistent with our gel shift assay data.

**Table 1:** ARX phosphorylation sites identified by MS

Identified sites			Corresponding human sites	Target	Peptide confidence			Sequence motif
Ctrl 1	Ctrl 2	CA 3			Ctrl 1	Ctrl 2	CA 3	
18	18		18S	S	high	high	o	ERPECKsKSPTLL
20	20		20S	S	high	high	o	PECKSKsPTLLSS
22	22		22T	T	high	high	o	CKSKSPtLLSSYC
25	25		25S	S	middle	high	o	KSPTLLsSYCIDS
26	26		26S	S	middle	high	o	SPTLLSsYCIDSI
31	31		31S	S	high	high	o	SSYCIDsILGRRS
	37		37S*	S	-	high	-	SILGRRsPCKMRL
		49	49S	S	-	o	low	LLGAAQsLPAPLA
		56	56S	S	-	o	low	LPAPLAsRADQEK
	67		67S*	S	-	low	-	EKAMQGsPKSSSA
142			141G	S	high	-	o	PGERQDsAGAVAA
		173	174S*	S	-	-	high	SISRSKsYRENGA
		266	267P	S	o	o	high	KEPRRCsVATTGT
		269	270A	T	o	o	high	RRCSVAtTGTVAA
		270	271T	T	o	o	high	RCSVATtGTVAAA
		272	273A	T	o	o	high	SVATTGtVAAAAA
	286		284T	T	o	high	o	AAAAVAtEGGELS
292	292	292	290S	S	high	high	middle	TEGGELsPKEELL
	312	312	310S	S	o	high	low	GKDGEDsVCLSAG
316	316	316	314S	S	high	high	high	EDSVCLsAGSDSE
319	319	319	317S	S	high	high	middle	VCLSAGsDSEEGL
	321	321	319S	S	o	high	low	LSAGSDsEEGLLK

Ctrl, Control experiments No.1 and No. 2.

CA, Calyculin A-treated experiment No. 3.

-, not covered by MS.

o, covered but no phosphate group detected.

* Reported by Mattiske *et al.* [23].

### PKA phosphorylates ARX at Ser^266^

**Figure 2:**
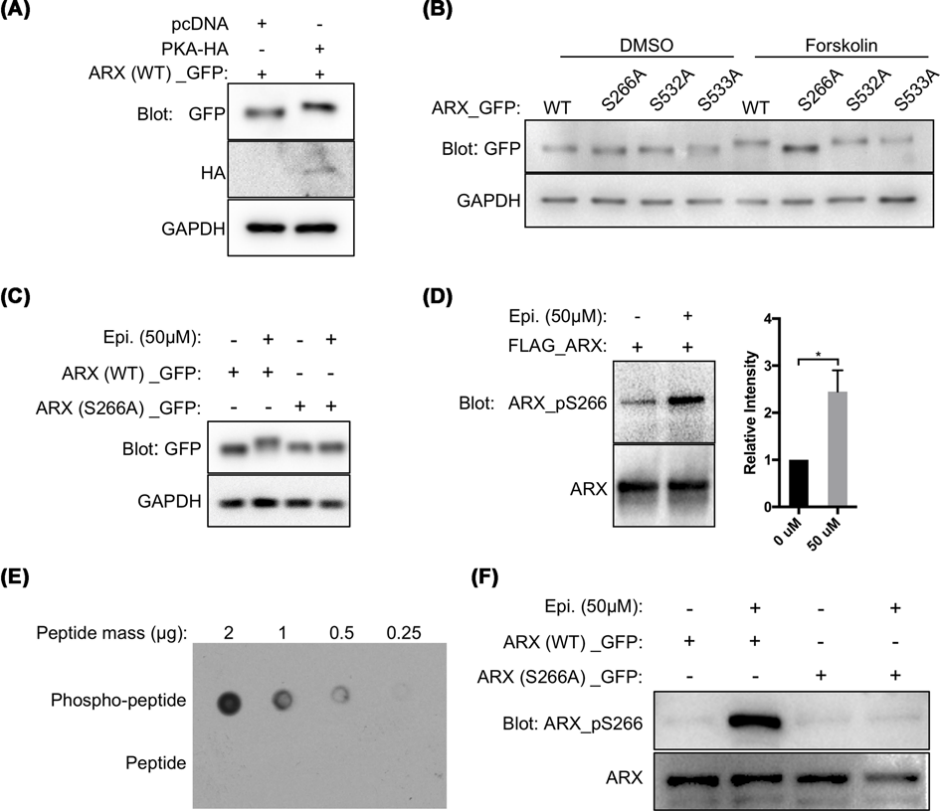
PKA phosphorylates S266 on ARX Western blots of 293T cells transiently transfected with a WT or mutant ARX expression vector. (**A**) Cells were co-transfected with control pcDNA vector or one expressing PKA. Cells co-expressing PKA and ARX show phosphorylated ARX when compared with cells expressing ARX alone. (**B**) Cells treated with DMSO or 50 μM Forskolin also showed evidence of phosphorylation. When the ARX^S532A^ and ARX^S533A^ mutant constructs were transfected, with or without Forskolin, the banding pattern was similar to WT ARX indicating similar phosphorylation patterns. In contrast, the expression of the ARX^S266A^ constructed resulted in no upper band in response to Forskolin indicating S266 is a site of phosphorylation. (**C,D**) 293T cells treated with 50 μM of epinephrine. Slower migrating band are presented in the WT ARX group but not with ARX^S266A^ (C). A S266 site specific phosphorylation antibody shows the increasing signal intensity with S266 phosphorylation (*P < 0.05, *n* = 3) (D). (**E,F**) Characterization of the ARX Ser^266^ phosphorylation site-specific antibody. Decreasing amounts of phosphorylated (CLKEPRRCp**S**VATTGTV) (upper row) or unphosphorylated (CLKEPRRC**S**VATTGTV) (lower row) ARX peptide including S266 were blotted on a nitrocellulose membrane. The S266 phosphorylation antibody only detected the phosphorylated peptide in the dot blot assay (E). Similarly, Western blots for eGFP tagged full-length ARX expressed by HEK 293T cells, treated with or without epinephrine (50 μM) for 1 h as indicated, revealed the S266 phosphorylation site-specific antibody only detects the S266 phosphorylated ARX in epinephrine treated cells, but not the site mutated ones (F). Similar levels of total ARX are detected with the αARX antibody (R&D Systems) (bottom row).

We next sought to identify the kinases responsible for ARX phosphorylation. Potential kinase(s) were first identified using motif-based online prediction tools (GPS [31–33], NetPhosK [34,35]). To validate these *in silico* data, we next selected kinases identified in both databases with a relatively high score for further study (Supplementary Table S1). *In vitro* kinase assays with purified ARX protein were performed using the HotSpot platform from RBC (Table 2). Six kinases (Aurora A, CDK4, CDK5, GSK3β, CK2α, and PKA) were tested. Among these six kinases, CK2α, PKA, and CDK4/cyclin D1 were found to have relative kinase activities, compared with the corresponding kinase’s RBC standard substrates, which represent 100% kinase activity, of 896, 120, and 45% on ARX, respectively. Aurora A, CDK5, and GSK3β showed little capacity to phosphorylate ARX. To further establish whether ARX is phosphorylated by these kinases, we co-expressed ARX with CK2, PKA, ERK1, CK1, PKC, PKG, GSK 3, CDK1, 2, 4, 5, and 6, or expressed ARX and treated the cells with the respective kinase inhibitor, then performed phosphorylation-dependent gel shift assays. Only the PKA showed a significant shift (Figure 2A and data not shown).

**Table 2:** Relative kinase activity on ARX*

Kinase		Aurora A	CDK4/Cyclin D1	CDK5/p35	CK2a	GSK3b	PKA
Substrate							
**ARX (1 μM)**	**1**	13.67	44.88	3.98	944.49	6.34	125.28
	**2**	13.25	46.34	4.16	921.34	6.61	126.45
	**3**	12.86	43.12	3.83	822.68	7.02	109.26
	**Average**	13.26	44.78	3.99	896.17	6.66	120.33
**Buffer**	**1**	1.30	−5.79	−0.29	18.63	−0.01	1.75
	**2**	0.60	−1.41	0.01	2.49	−0.13	−0.50
	**3**	−1.68	−1.57	−0.35	−0.68	0.29	−1.84
	**Average**	0.07	−2.92	−0.21	6.82	0.05	−0.20
**Standard**	**1**	89.05	106.70	103.65	118.35	100.31	99.63
	**2**	100.35	98.38	95.71	88.59	95.54	102.09
	**3**	110.60	94.91	100.65	93.06	104.15	98.28
	**Average**	100.00	100.00	100.00	100.00	100.00	100.00

*All kinase activities are presented as percentage of the standard substrate. Each reaction was performed in triplicate.

We next ranked the predicted sites on ARX based on their GPS scores (Supplementary Table S1, Table 3) and the *in vitro* kinase assays (Table 2). Among the PKA scores, S266 received the highest score and was uniquely identified by MS in the CA-treated group (Table 1). This suggested a reversible phosphorylation site that could be regulated by physiologic conditions. To establish if PKA can phosphorylate ARX at S266, we first evaluated ARX after treating 293T cells with Forskolin, a PKA activator [36–38]. A clear shift of WT ARX (ARX^WT^) protein band was observed (Figure 2B). To confirm the specificity of this result, we generated an ARX expressing construct with an S266A mutation (ARX^S266A^), eliminating the capacity for ARX to be phosphorylated at this site. As predicted, the mutant protein showed no gel shift indicating it could not be phosphorylated. We also generated ARX^S532A^ and ARX^S533A^ constructs, other predicted PKA phosphorylation sites based on the *in-silico* analysis. Gel shifts for these mutant proteins were still observed, indicating PKA likely does not phosphorylate at these sites (Figure 2B).

**Table 3:** Top sites phosphorylated by PKA and CK2α

PKA				CK2α			
Position	Code	Score*	Cutoff^†^	Position	Code	Score	Cutoff
266	S	7.429	4.405	319	S	15.197	9.894
532	S	7.333	6.283	220	T	12.159	9.894
507	T	7.333	6.283	56	S	10.483	9.894
67	S	7	6.283				
72	S	6.667	6.283				

* Score: Phosphorylation potential was calculated by the GPS 3.0 algorithm. Higher values represent a greater potential for phosphorylation at the designated residue.

† Cutoff: calculated minimum thresholds were established through standardized methods (GPS 3.0). If the predicted value > cut-off value, the residue is considered as statistically likely to be phosphorylated.

Epinephrine and glucagon are known activators of PKA through binding to a G protein-coupled receptor on the target cells [39]. We utilized this known pathway by treating 293T cells, transfected with an ARX expressing construct, with epinephrine to determine whether ARX phosphorylation would be enhanced. A phosphorylated form of ARX^WT^ was isolated from the epinephrine-treated cells (Figure 2C, upper band), whereas the cells transfected with the ARX^S266A^ expressing construct showed no band shift (Figure 2C). Increased S266 phosphorylation was also detected with the phosphorylation site-specific antibody, further supporting S266 as the phosphorylated residue (Figure 2D–F). Besides, PKA activity is suppressed by PP1/PP2A phosphatases, which are CA-sensitive protein phosphotases [40]. As a complimentary experiment, our gel shift assay with the phosphatase inhibitor CA treatment indicated phosphorylation to be responsible for the shift in protein size (Figure 1B).

Together, these data suggest S266 is a putative PKA phosphorylation site, possibly the only PKA phosphorylation site in ARX. ARX may also be phosphorylated by CK2α and CDK4/cyclin D1, but not Aurora A, CDK5/p35, and GSK 3β.

### The subcellular localization and transport of ARX is independent of the identified phosphorylation sites

**Figure 3:**
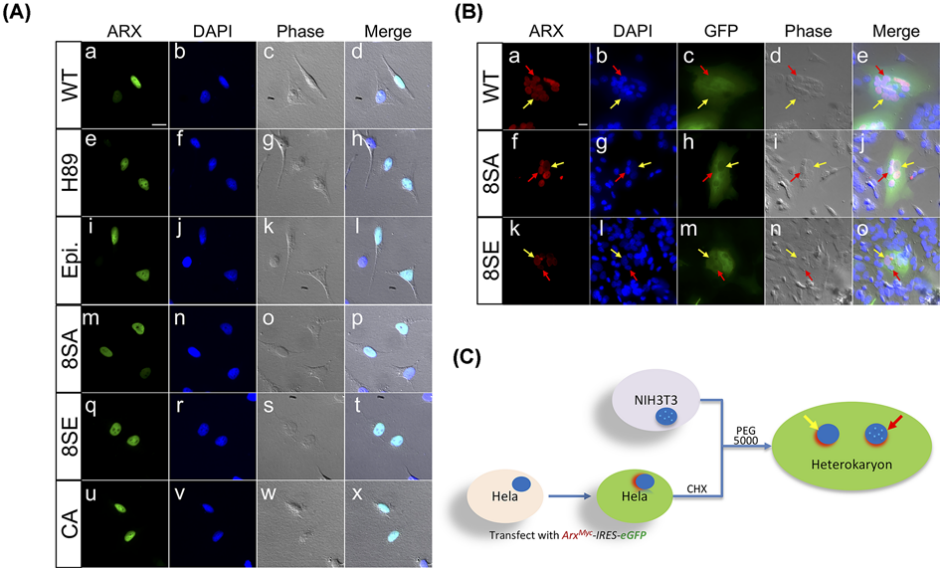
The subcellular localization and heterokaryon assay of ARX mutations (**A**) HeLa cells were transfected with WT or mutant ARX expressing constructs, with or without treatment (as labeled), fused with an eGFP tag, all localized in the nucleus (Green: WT or mutant eGFP-ARX; Blue: DAPI). (**B**) The heterokaryon of HeLa cells and NIH3T3 cells. HeLa cells (recognized by the diffused DAPI staining pattern, yellow arrow) were transfected with WT or mutant *Arx^Myc^-IRES-eGFP* constructs as labeled. These cells were fused with non-transfected NIH3T3 cells (spotted DAPI staining pattern, red arrow). HeLa cells were treated with CHX for 3 h before cell fusion to inhibit new protein synthesis. The WT and mutant ARX^myc^ proteins were immunolabeled with anti-myc antibody (red: a, f and k). GFP (Green: c, h and m) along with bright field (2× phase contrast: d, i and n) to define the heterokaryon. Both ARX^WT^ and ARX^mut^ were able to be transported from the donor cells to the recipient cell nucleus. Scale bars in the upper left panel is 20 μm for all images. (**C**) A schematic of the experiment forming heterokaryons between transfected HeLa cells and NIH3T3 cells. Nuclei are indicated with (NIH3T3 cells, red arrow) or without (HeLa cells, yellow arrow) blue bright dots; nucleus with red shadow represent the cells expressing ARX; the cytosol (green) expresses GFP, indicating the cells transfected with plasmids (defining the heterokaryon).

Having determined that ARX is phosphorylated by PKA at S266, we next asked whether its subcellular localization is affected by PKA. To investigate the cellular localization based on phosphorylation, we treated HeLa cells with the PKA inhibitor H89 or the PKA activator epinephrine (Figure 3A,a–l). Unexpectedly, they did not show a change in the subcellular localization of ARX.

Given that the phosphorylation by PKA did not affect the ARX subcellular location, we considered other identified sites for a role in localizing ARX to the nucleus. We generated the loss of function (LOF) and gain of function (GOF) constructs by replacing individual serines with alanine (LOF) or glutamine (GOF) at multiple sites (Supplementary Table S2). Including the S266A/E mutations, these mutant constructs showed normal subcellular localization of ARX (data not shown). We next considered whether some combination of phosphorylation mutations might dictate protein localization. Based on the MS results, the *in vitro* kinase assay data, the prediction scores, the previous reports and the relative position to functional domains, we selected eight sites (S49, S173, S266, S292, S312, S316, S319, S321) to generate multisites LOF and GOF mutation constructs. The ARX^8SA^ (LOF) or ARX^8SE^ (GOF) constructs were generated with an in-frame GFP tag. Surprisingly, both the ARX^8SA^ and ARX^8SE^ constructs showed normal nuclear localization (Figure 3A,m–t), and the subcellular localization of ARX was not affected by the treatment with the protein phosphatase inhibitor CA (Figure 3A,u–x).

Next, since we have established that ARX shuttles between the cytoplasm and nucleus (unpublished data), we tested whether this function might be regulated by phosphorylation. To accomplish this, we generated the *Arx*^*WT_myc*^-*IRES-eGFP, Arx^8SA_myc^-IRES-eGFP*, and *Arx*^*8SE_myc*^-*IRES-eGFP* constructs and performed heterokaryon assays [41]. Again, both of the mutant forms of ARX were capable of transporting from the donor cells (HeLa cells) to the acceptor cell nuclei (NIH3T3 cells) (Figure 3B,C).

### The *in vitro* and *in vivo* functional tests shows no changes of ARX phosphorylation mutants

**Figure 4:**
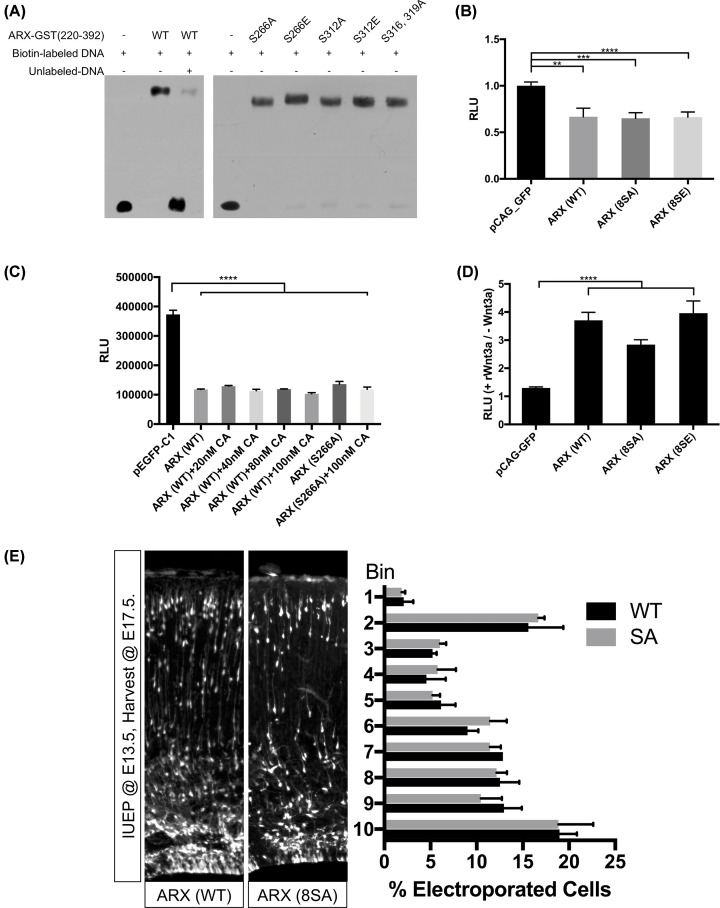
Functional tests of the ARX with different phosphorylation status (**A**) EMSAs demonstrate that ARX-GST (220–392) can bind to double-stranded oligonucleotides that contain the ARX binding site; all mutations tested had the same binding activity as WT ARX (protein loading was carefully controlled to enable comparisons between constructs with 5 μg of protein loaded in each lane). (**B**–**D**) Quantification of the luciferase reporter assays in HEK 293T cells co-transfected with constructs as indicated (RLUs). (B) Ebf3-luciferase activity is significantly repressed by ARX (WT and mutant), with no significant difference between WT and mutant constructs transfection. (C) Cells were treated with indicated concentration of phosphatase inhibitor CA for 30 min. The treatment did not perturb the repressive transcriptional activity of ARX to Lmo1-luciferase activity. (D) Top-flash reporter assay indicated both WT and mutant ARX have the same ability to activate the Wnt signaling pathway (**P < 0.01, ***P < 0.001, ****P < 0.0001; *n* = 3). (**E**) Representative images of the embryonic cryosections from *Arx^flox/flox^ Ai14^hom^* mice electroporated with *Nestin:Arx^WT or 8SA^-IRES-Cre*. The cortex was divided into ten equal bins for the quantification. Cells electroporated with a WT construct or the 8SA construct showed a similar positional organization (*n* = 3 for each genotype).

To establish whether phosphorylation is necessary for the transcriptional activity of ARX, we next performed DNA binding and reporter assays as previously described [12,13]. DNA binding was established by EMSA using purified ARX fragment 220–392 with or without mutations. LOF and GOF mutations at S266 in ARX bound DNA with equal affinity as WT ARX protein. Similarly, both LOF and GOF mutations at S312, S316, and S319 had no impact on DNA binding (Figure 4A). These three sites are the closest sites to the NLS2/3 among the sites identified by MS. Even more, S319 is the site got the highest predicted score of CK2α phosphorylated sites (Table 3), and CK2α presented the highest activity in *in vitro* kinase assays (Table 2). These data suggest the phosphorylation status of ARX at these identified sites does not appear to influence target DNA binding.

ARX is known to function mainly as a transcriptional repressor. Reporter assays have been established to measure this activity [24]. The multisite LOF and GOF mutation constructs, ARX^8SA^ and ARX^8SE^, respectively, were both tested and found to have normal transcriptional repressive activity (Figure 4B). Given that mutating these eight serine sites resulted in no changes in the repressive transcriptional activity for ARX, we also tested the transcriptional activity of ARX with many additional single-site mutations outside of the eight sites in the multisite mutants. These included S18A/E, S20A/E, T22A/E, S208A/E, T335A/E, S359A/E, and S408A/E; all of which showed normal transcriptional repressive activity (data not shown). We next tested the transcriptional repression activity of WT ARX from cells treated with different concentrations of CA. Unexpectedly, the transcriptional activity did not show any dose associated trend, and even the group treated with the highest concentration (100 nM) of CA had the same activity as untreated cells, ARX S266A has the same effect (Figure 4C). Consistent with these data, the TOP-flash assay, assessing the transcriptional activation function of ARX in Wnt signal pathway, also did not show any changes with the ARX^8SA^ or ARX^8SE^ mutations [13] (Figure 4D).

Finally, we recognized all of the data we had generated were *in vitro*. Given that the disfunction of ARX in the cortex could induced the defect of the migration of neuron cells, also to ensure our *in vitro* data were not an artifact of culture conditions, we performed a series of *in vivo* experiments to test whether the ARX^8SA^ or ARX^8SE^ mutations would affect the radial migration of neural progenitor cells (NPCs) in the developing mouse brain [12]. Expectedly, these mutations had no impact on neuronal migration (Figure 4E).

## Discussion

The transcription factor ARX plays essential roles in development [24]. Mutations in *Arx* are associated with a variety of structural and functional neurologic disorders [21]. Thus, a more completed understanding as to how ARX functions will provide valuable insights into many related human disorders. Herein we investigated the hypothesis that phosphorylation, a common modulator of protein function [2,13], might be regulating ARX. We confirmed that ARX is post-translationally modified by phosphorylation and assessed the role of ARX phosphorylation in regulating its cellular functions. Twenty-two phosphorylation sites in ARX were identified along with several kinases that function to phosphorylate ARX at these sites.

We inferred from our data that two types of phosphorylation exist in ARX. The first, including S292, S312, S316, and S319, are believed to be ‘constant sites’. The serine phosphorylation at these sites was detected by MS in both control and experimental cells with high confidence. The second group was considered as the potential regulatory sites as they only appeared (S49, S56, S266, T269, T270, T272) or disappeared (S18, S20, T22, S25, S26, S31) after the treatment with CA. As CA inhibits the activity of protein phosphatases PP1 and PP2A [28], it is easy to understand the sites only showed in the CA-treated group. While the chemical treatment may also create a selective pressure on the cells, that could be a potential explanation that some sites were identified only after CA treatment.

Although the phosphorylation of the ‘constant sites’ did not change under the tested conditions, it is not sufficient to conclude that these sites are universally ‘constant’. Signaling pathways’ activity levels are variable between different cell lines. Similarly, the regulatory sites here may be ‘constant’ under other conditions. We used HEK 293T cells because it represents a commonly used mammalian cell line, and it is efficiently transfected to get relatively high protein yield for IP and MS.

It was interesting to note that all of the ‘constant sites’ were located adjacent to the 5′ of the homeodomain, which is a highly conserved domain through evolution [42]. We speculate these sites might be necessary for normal ARX function. Alternatively, the regulatory sites may represent sites that can quickly respond to stimulations or other development events.

Furthermore, most phosphorylation sites we identified were clustered in two regions: aa 18–67 and aa 266–321. However, no phosphorylation sites were detected in the C-terminus (aa 322–564). Recent studies have identified a C-terminal region (aa 472–562) of ARX that is bound by PICK1 (Protein interacting with C kinase 1), a scaffold protein which is known to facilitate phosphorylation, while PRKCA phosphorylates ARX at S174 [23]. We have also found that the C-terminus of ARX (aa 431–564) binds to the Armadillo domains of the β-catenin and also to P300 to regulate the Wnt signaling pathway [13]. Although we used the most common enzyme, trypsin, for protein digestion prior to MS analysis, we are aware of at least 11 serines that would likely not been identified as potential phosphorylation sites as they lack digestion sites (aa 40–43, 1 serine; aa 203–257, 1 serine; aa 382–475, 9 serines). Of note, another study seeking to identify phosphorylation sites in ARX did include aa 382–475, but no phosphorylation sites were detected [23]. Interestingly, several of the phosphorylation sites we identified in ARX (e.g. S18, S37, S67, S174) were also identified by high throughput method [43]. All together, we systematically identified multiple new phosphorylation sites on ARX, provided a brief classification of the sites and confirmed that ARX is phosphorylated primarily at the N-terminus. We postulate the C-terminus binds molecular partners for functions that remain to be elucidated.

We also sought to identify the kinase(s) that phosphorylate ARX, as they also represent potential directions to consider modulating functions during development. To this end, we took several approaches, including a direct kinase screen. Three kinases, Aurora A, GSK 3β, and CDK5/p35, were confirmed not to phosphorylate ARX in our assays. Three kinases, PKA, CK2α, and CDK4/cyclin D1, were identified, as candidates, to phosphorylate ARX. Among the three candidate kinases, we confirmed PKA to phosphorylate ARX during PKA overexpression or PKA stimulation by Forskolin or Epi, although both may activate other kinases and thus indirectly phosphorylate or induce other modifications of ARX leading to the observed band shifts in Figures 1B and 2A–C. Interestingly, the S266 site, identified as the unique PKA phosphorylation site on ARX, only exists in mice making this site a less attractive target to play a critical role in an essential function.

Additionally, CK2α is a universally highly expressed kinase, raising the possibility that the site(s) it phosphorylates could be constitutively phosphorylated. CK2 has been reported to regulate the subcellular localization [44] or the DNA binding function of homeodomain proteins [45]. These characteristics of CK2 suggested that CK2 as a likely candidate to modulate ARX function. Previous studies found that ARX could repress *cdkn1c* [46], whose gene product is a tight-binding inhibitor of CDK4/cyclin D2 complex during the cell cycle [47–49]. This interaction suggested that ARX and CDK4 might form a complex with DNA creating a feedback loop to regulate the function(s) or the expression level of each other through the cell cycle. However, it remains possible that under different specific conditions, some of the kinases we excluded could be functional.

The results of our functional *in vitro* luciferase assay using HEK293T cells coincide with the research from Mattiske et al., 2018 [23]. Their studies suggest that the transcriptional activity of ARX on *Lmo1* promoter was not changed by the phospho-null mutation of S174 (S173 in mouse). In a similar experiment, we tested seven more sites and found a comparable effect. Although they confirmed that the transcriptome of alpha-TC cells was changed after the over expression of the mutant ARX, our IUEP *in vivo* study, which was performed in brains has very high expression level of ARX, still show no significant changes caused by the phospho-null mutations. Future work will focus on exploring other functions, together with other molecular partners, affected by the phosphorylation sites in certain context.

In summary, we have shown that ARX is phosphorylated at multiple sites, and multiple kinases could be responsible for the phosphorylation of ARX, making this post-translational modification an excellent mechanism to modulate the various functions served by this one multifunctional protein. Although the functional tests we performed did not show any significant changes with the mutation of the phosphorylation sites, future studies, using the data from the present study, will likely elucidate the mechanism of ARX phosphorylation and how ARX is able to module its many functions through protein modification.

## Supplementary Material

Supplementary Figure S1

Supplementary Tables S1-S2

## Abbreviations

AD: Aristaless domain
ARX: Aristaless-related homeobox protein
CA: Calyculin A
CDK4: cyclin dependent kinase 4
CHX: cycloheximide
CIAP: calf intestinal alkaline phosphatase
CK2α: Casein Kinase 2α
EMSA: electrophoretic mobility shift assay
FGF-2: fibroblast growth factor 2
GOF: gain of function
GSK 3β: Glycogen Synthase Kinase 3β
HD: homeodomain
IP: immunoprecipitation
IUEP: *In utero* electroporation
LOF: loss of function
MS: Mass Spectrometry
mut: mutation
NLS: nuclear localization sequence
NLS2: nuclear localization sequence 2
NPC: neural progenitor cell
OD: octapeptide domainpolyethyleneimine
PFA: paraformaldehyde
PKA: protein kinase A
PKC: protein kinase C
PMSF: phenylmethylsulfonyl fluoride
PP1: protein phosphatase 1
PP2A: protein phosphatase 2A
RBC: Reaction Biology Co.
SDS: sodium dodecyl sulfate
SEM: standard error of the mean
WT: wildtype
