# Supplementary material for: Identification and validation of the phosphorylation sites on Aristaless-related homeobox protein
Source: Biosci Rep. 2020 Jul 15;40(7):BSR20194513. doi: 10.1042/BSR20194513 (PMC7364481; doi:10.1042/BSR20194513)
Supplement: Supplementary Figure S1 [file BSR-2019-4513_supp.pdf]

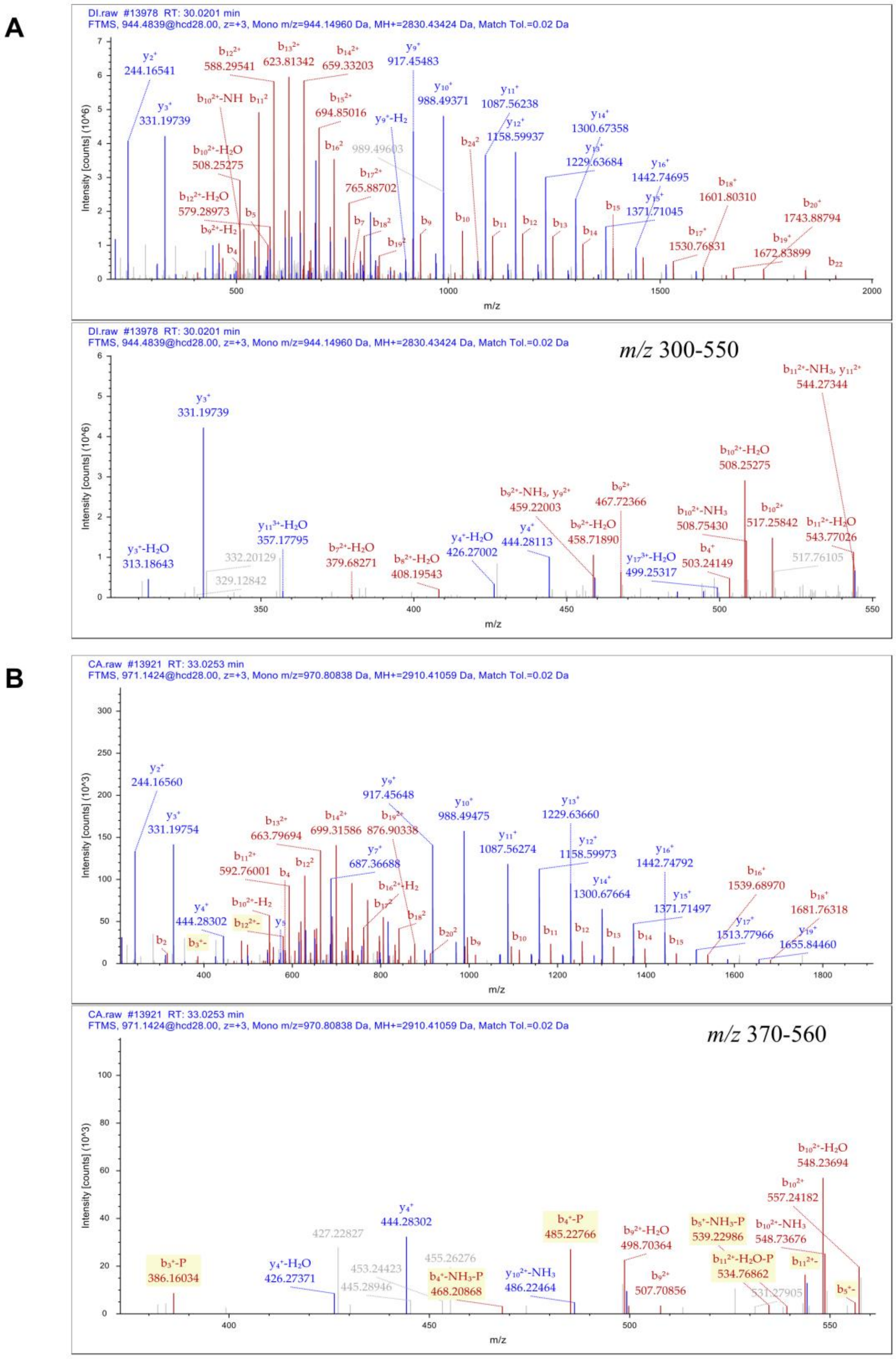

Figure S1: ARX Serine 266 phosphorylation was only detected in Calyculin A treated HEK 293T cells by Mass Spectrometry.
